# Anti-TNF therapy for inflammatory bowel disease in patients with neurodegenerative Niemann-Pick disease Type C

**DOI:** 10.12688/wellcomeopenres.16986.1

**Published:** 2022-01-11

**Authors:** Isabelle Williams, Sumeet Pandey, Wolfram Haller, Hien Quoc Huynh, Alicia Chan, Gesche Düeker, Ruth Bettels, Laurent Peyrin-Biroulet, Chinenye R. Dike, Catherine DeGeeter, David Smith, Nada Al Eisa, Nick Platt, Thorsten Marquardt, Tobias Schwerd, Frances M. Platt, Holm H. Uhlig

**Affiliations:** 1 Translational Gastroenterology Unit, University of Oxford, Oxford, UK; 2 Birmingham Children's Hospital, UK, Birmingham, UK; 3 Department of Paediatrics, Stollery Children's Hospital, University of Alberta, Alberta, Canada; 4 Division of Clinical Genetics, Stollery Children's Hospital, University of Alberta, Alberta, Canada; 5 University Children's Hospital Bonn, Bonn, Germany; 6 Children's Hospital Munster, Munster, Germany; 7 Inserm U1256 NGERE, 8 Lorraine University, Vandoeuvre-les-Nancy, France; 8 Department of Gastroenterology, Nancy University Hospital Center, Vandoeuvre-les-Nancy, France; 9 Stead Family Department of Pediatrics, University of Iowa, Iowa, USA; 10 Department of Pharmacology, University of Oxford, Oxford, UK; 11 College of Medicine, King Saud bin Abdulaziz University for Health Sciences, Riyadh, Saudi Arabia; 12 Department of Paediatrics, Dr. von Hauner Children’s Hospital, Munich, Germany; 13 Department of Paediatrics, University of Oxford, Oxford, UK; 14 Biomedical research centre, University of Oxford, Oxford, UK

**Keywords:** Niemann-Pick disease Type C1

## Abstract

**Background:**  Blockade of tumour necrosis factor (anti-TNF) is effective in patients with Crohn’s Disease but has been associated with infection risk and neurological complications such as demyelination. Niemann-Pick disease Type C1 (NPC1) is a lysosomal storage disorder presenting in childhood with neurological deterioration, liver damage and respiratory infections. Some NPC1 patients develop severe Crohn’s disease. Our objective was to investigate the safety and effectiveness of anti-TNF in NPC1 patients with Crohn’s disease.

**Methods:** Retrospective data on phenotype and therapy response were collected in 2019-2020 for the time period 2014 to 2020 from patients in the UK, France, Germany and Canada with genetically confirmed NPC1 defects and intestinal inflammation. We investigated TNF secretion in peripheral blood mononuclear cells treated with NPC1 inhibitor in response to bacterial stimuli *.*

**Results:** NPC1 inhibitor treated peripheral blood mononuclear cells (PBMCs) show significantly increased TNF production after lipopolysaccharide or bacterial challenge providing a rationale for anti-TNF therapy. We identified 4 NPC1 patients with Crohn’s disease (CD)-like intestinal inflammation treated using anti-TNF therapy (mean age of onset 8.1 years, mean treatment length 27.75 months, overall treatment period 9.25 patient years). Anti-TNF therapy was associated with reduced gastrointestinal symptoms with no apparent adverse neurological events. Therapy improved intestinal inflammation in 4 patients.

**Conclusions:** Anti-TNF therapy appears safe in patients with NPC1 and is an effective treatment strategy for the management of intestinal inflammation in these patients.

## Introduction

Niemann-Pick disease Type C (NPC) is an autosomal recessive lysosomal storage disorder presenting most typically throughout childhood or adolescence ^1^. Variants in the *NPC1* gene account for 95% of the genetic defects in patients with NPC ^1^. Patients experience progressive neurological impairment with reduced muscle tone, seizures, speech impairment and early onset dementia as well as hepatosplenomegaly, interstitial lung disease with recurrent pneumonia ^1^. 

Around 7% of patients with NPC1 develop inflammatory bowel disease (IBD), in particular Crohn’s disease with weight loss, diarrhoea, perianal fissures and fistula formation ^2^. The NPC1 defect disrupts auto-phagosome maturation which impairs antibacterial responses towards *Salmonella typhimurium* and *Adherent-invasive E coli (AIEC)* ^2^. Mice with NPC1 deficiency can develop mild intestinal inflammation ^3^.

In paediatric and adult patients with Crohn’s disease anti-TNF agents (such as infliximab and adalimumab) are standard of care to induce and maintain remission ^4, 5^. However, anti-TNF treatment can cause neurological side effects ^6^ such as demyelination ^7, 8^ and non-demyelinating central nervous system (CNS) inflammation including vasculitis ^9, 10^. Since NPC1 patients could be particularly vulnerable to adverse neurological events due to their underlying progressive neurological disease we investigated if anti-TNF is a safe and effective treatment strategy for intestinal inflammation in NPC patients.

## Methods

### Study design

In this retrospective cohort study, we identified patients with NPC1 and intestinal inflammation by contacting specialist centres involved in their care. We recruited patients with NPC1 and intestinal inflammation who had been on anti-TNF agents as part of their management. Data collection took place in 2019 –2020 with data collection from 2014 until 2020. Selection bias was reduced by the use of pre-determined inclusion criteria, and as a consequence, patients with incomplete datasets were excluded. Data was collected from historical medical records to calculate wPCDAI scores to reduce memory bias.

### Patient cohort

Patients with genetically confirmed NPC1 and confirmed intestinal inflammation (endoscopically and histologically) treated with anti-TNF agents were identified by contacting specialist facilities responsible for their care in Europe and Canada. We identified 5 patients and retrospective data was collected. Data was obtained from local hospital notes by the patients’ lead clinician. Out of five patients, one was excluded due to incomplete data being available. 

De-identified patient information was provided by the clinicians responsible for their care.

Information on previous treatment and responses (dates of treatment, reason for stopping treatment and pre and post treatment severity of abdominal pain, stools per day, perianal disease and presence extraintestinal manifestations) were obtained in addition to response to anti-TNF therapy. The mathematically weighted paediatric Crohn’s disease activity index (wPCDAI) ^11^ was calculated at baseline, 4 weeks, 10 weeks, 6 months and a year after starting anti-TNF agents. A change in wPCDAI of >17.5 was used to indicate a response to treatment with a wPCDAI <12.5 suggesting remission, wPCDAI > 40 suggesting moderate disease and wPCDAI > 57.5 suggesting severe disease ^11^. Potential side effects including unexpected changes in neurological function and infectious complications (e.g. increased frequency of respiratory infections) since starting anti-TNF therapy were recorded.

### In vitro Infection model

Peripheral blood mononuclear cells (PBMCs) from healthy volunteers supplied from the NHS blood bank as leukocyte cones were obtained via the Oxford GI biobank.

PBMCs were separated from whole blood by using a Ficoll gradient. PBMCs (400,000 cells per condition) were either treated with 2ug/ml U18666A (Sigma Aldrich) drug for 24 hours or were left untreated. After U18666A treatment, PBMCs (400,000/well) were either stimulated with lipopolysaccharide (LPS) (200 ng/ml) orinfected with *Salmonella typhimurium* (Multiplicity of infection (MOI): 10) or Adherent invasic E. Coli ( *AIEC)* (MOI: 10). Supernatant was collected and frozen at 0, 2, 4 and 6 hours after stimulation. TNF quantification was performed using an eBioscience ELISA kit (catalogue number BMS223–4) as per manufacturer’s protocol.

### Statistical analysis

Data analysis was performed using GraphPad Prism 9 software (GraphPad software, Inc., San Diego, CA). R is an open access alternative. Statistical significance was calculated using a Mann-Whitney U test or ANOVA for multiple test comparison. P < 0.05 were considered significant (* *p* < 0.05, ** *p* < 0.01, *** *p* < 0.001, **** *p* < 0.0001).

### Ethics

Written informed consent was obtained locally by the patients’ lead clinician from the patients or their guardians for deidentified data to be used for research purposes. Patient data were collected as part of the Oxford IBD cohort study and a sub-project to investigate rare diseases (The Oxford Gastrointestinal Illness Biobank IRAS ID 210441). Anonymous patient data were analyzed. Healthy donor samples were obtained via Oxford GI illnesses biobank.

## Results

### Small molecule inhibition of NPC1 increased TNF levels

**Figure 1.**
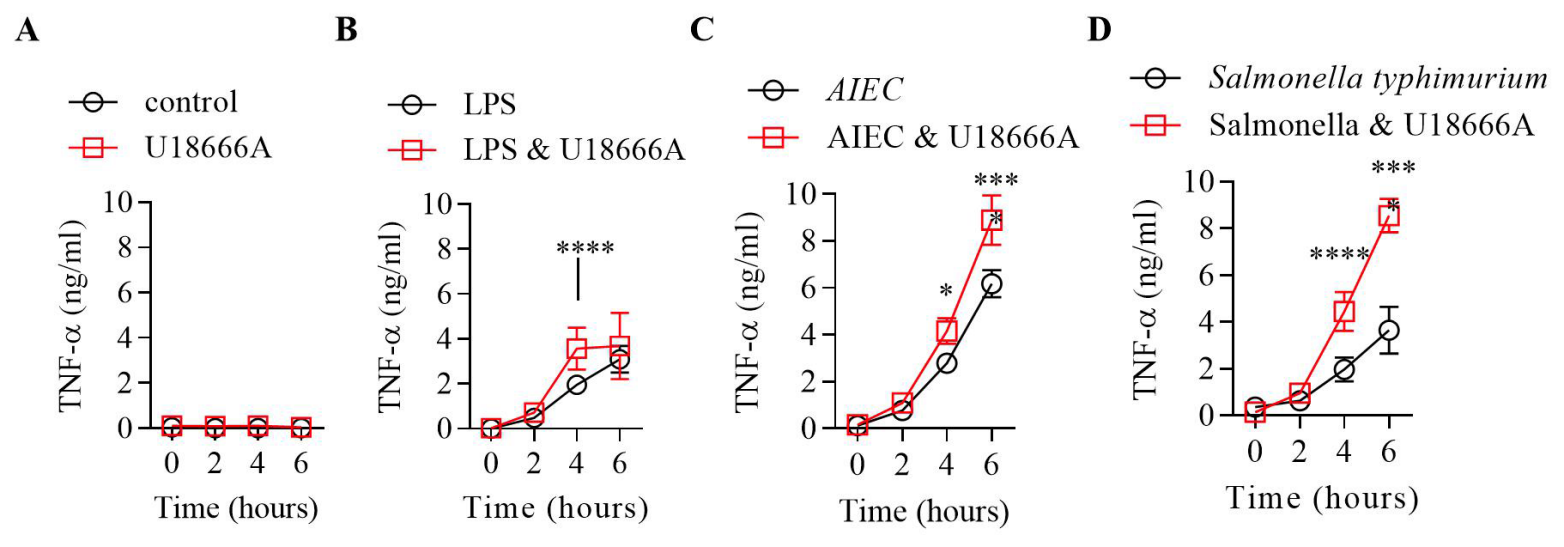
Tumour necrosis factor (TNF) quantification after bacterial exposure in U-drug treated or control PBMCs. Peripheral blood mononuclear cells (PBMCs) (400,000) were either treated with U-drug for 24 hrs or left untreated. A-D: Post U-drug treatment PBMCs were stimulated with LPS, *Salmonella typhimurium* and *AIEC* and supernatants were collected at 0-, 2-, 4- and 6-hour time points for TNF quantification by ELISA. Statistical significance was determined using two-way ANOVA, multiple comparisons; Bonferroni’s multiple comparisons test (*p<0.01 and **** p<0.0001).

We investigated whether pharmacological inhibition of NPC1 with U18666A ^2^ caused a TNF associated inflammatory cytokine response. Healthy volunteer PBMCs were incubated with U18666A ^2^ before being stimulated with LPS, *AIEC* or S *almonella*. At baseline (0 hour) we did not observe any significant differences in TNF levels between U18666A treated and control cells ( Figure 1A) ^12^. After stimulation with LPS we found significantly higher TNF levels at the 4-hour time point only ( Figure 1B). In contrast, stimulation with *AIEC* and S *almonella* resulted in significantly higher level of TNF production at the 4- and 6-hour time points in U18666A treated cells compared to controls (* *p*<0.01, **** *p*<0.0001) ( Figure 1C&D). This suggests increased TNF production in response to LPS and gut bacteria in patients with NPC1 may contribute to mucosal inflammation.

### Anti-TNF treatment of NPC1 patients with Crohn’s disease (CD) like intestinal inflammation

**Figure 2.**
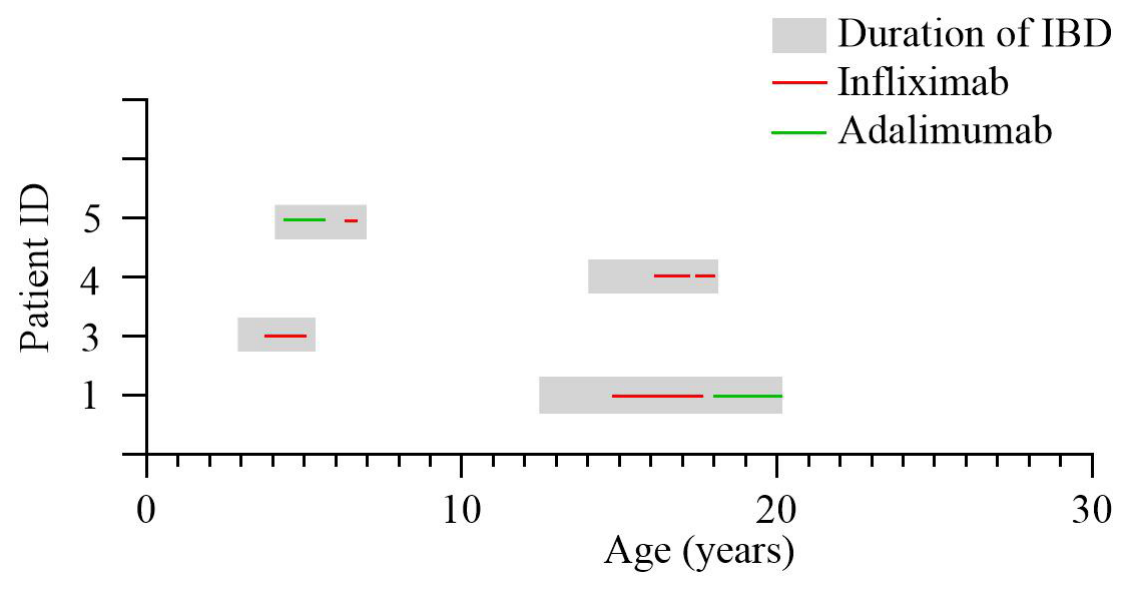
Anti-TNF (tumour necrosis factor) therapy in patients with *NPC1* genetic defect and Crohn’s disease (CD). The grey shaded area represents time from age of IBD diagnosis to age at most recent follow up data. Red line represents duration of infliximab therapy with green line representing adalimumab therapy. IBD=inflammatory bowel disease.

We identified 4 patients with NPC1 and Crohn’s disease-like intestinal inflammation who received anti-TNF therapy. Two patients presented with complex perianal disease, fistulation, pain and weight loss ( Table 1). Patient numbers 1 and 4 have been described previously and we provide updated results with treatment response here ^2^. The mean age of Crohn’s disease onset was 8.1 years with a mean treatment period of 27.75 months. Therapy used for control of intestinal inflammation was exclusive enteral nutrition (EEN) (n=1), local steroids (n=1), systemic steroids (n=4), 5-ASA (n=2), adalimumab (n=2), infliximab (n=4), methotrexate (n=1), anti-IL12/23p40 targeting ustekinumab (n=1) and the integrin antagonist vedolizumab (n=1). Two patients received both infliximab and adalimumab (patients 1 and 4) and patient 3 stopped infliximab and was switched to ustekinumab due to loss of effect before restarting both agents on family request. Complete remission was temporarily achieved in one patient (patient 1) using infliximab induction therapy. All other patients achieved a partial response (patients 2–4) ( Table 2). Two patients (patients 1 and 4) received adalimumab after loss of response (patient 1). Patient 4 was commenced on adalimumab first before switching to infliximab after loss of response to therapy. Infliximab trough-levels or anti-drug antibodies were not available. The duration of anti-TNF treatment ranged from 1.2 year to 5.4 years. The overall treatment period with anti-TNF was 9.25 patient years ( Figure 2). 

**Table 1.** Baseline characteristics of patients.

Patient ID	Sex	Age at IBD diagnosis (years)	Mutation	Intestinal symptoms	Endoscopic and histological findings	Paris Classification	Extra-intestinal manifestations
1	F	12.4	c.3019C>G (p.P1007A) and c.3731T>C (p.L1244P)	Diarrhoea, rectal bleeding, pain on defecation, perianal fissures, anorectal and vaginal fistula, arthritis	Aphthous lesions in the colon + fistulas Histology - inflammatory infiltrates, no granulomas.	A1bL2B1p	
2	F	2.9	compound heterozygous for c.2678dupT and c.3107C>T also heterozygous for c.29T>G	Rectal bleeding and diarrhoea	Severe left sided disease, inflammatory stricture of distal rectum, severe perianal skin tagging.	A1aB2B3pL2G1 ^#^	Respiratory infections, growth delay, neurological deterioration with swallowing dysfunction
3	F	13.6	c.2848G>A and c.423_424dupGA.	Diarrhoea with blood, weight loss	Mild colitis endoscopically with granulomas on biopsy	A1bL2B1G0	Progressive motor, speech and bulbar dysfunction with swallowing dysfunction and moderate OSA (uses CPAP). Wilms tumour age 4 (surgery and chemotherapy)
4	M	4.2	p.Ile1061Thr and p.Ser847Pro	Perianal pain, weight loss	Perianal skin tags and fissures, macroscopically normal colon and terminal ileum. Histology – IEL and granulomas	A1aB1pL1G0	Splenomegaly, no neurological symptoms at present

^#^ : unable to have complete assessment at colonoscopy due to disease severity. 1* represents patient 8 in Schwerd *et al*., 4 ^+^ patient case report previously published ^13^ IEL: intraepithelial lymphocytes

**Table 2.** Response to treatment of 5 patients with NP-C and Crohn’s-like disease.

Patient ID												
	EEN	Steroids, systemic	Steroids, local	5-ASA	IFX	ADA	Vedo	MTX	AZA / 6-MP	AB	Other	Side effects
1	-	I, partial	-	I, poor	I, complete, M, partial, loss of response after 2.5 years	M, partial	-	-	-	-	-	
2	I, poor	I, partial	-	-	M, partial	-	-	-	M, partial	M, partial	-	-
3	-	I, partial	-	I, poor	M, partial	-	-	-	M partial	M, partial	Ustekinumab (M, partial).	-
4	-	I, partial	I, poor	-	M, partial	M, partial	M partial	M, partial	-	-	2- hydroxypropyl-β-cyclodextrin	-

EEN = exclusive enteral nutrition, IFX = infliximab, ADA = adalimumab MTX = methotrexate, AZA = azathioprine, 6-MP = 6 mercaptopurine, AB = antibiotics. I= induction, M = maintenance therapy. Response classified as complete, partial or poor. Complete induction was defined as successful induction of remission, partial as improvement of disease activity and poor as little or no response. Maintenance treatment was classified complete if there was control of intestinal inflammation with no flares or complications, partial if there was good control of IBD activity and poor if there was little or no response.

### Anti-TNF therapy appears safe in patients with NPC1 and Crohn’s disease like intestinal inflammation

In all 4 patients who received anti-TNF therapy, over the 1 year of follow up, we did not observe any adverse neurological events or worsening of neurological function suggesting anti-TNF therapy does not accelerate neurological decline in this patient group. Furthermore, there was no increase in respiratory complications or other serious infection in patients reported in this cohort at 1-year of follow up. Neurological decline was seen in some patients after the first year follow up period however, clinicians felt this was consistent with NPC1 progression. Patient 2 died whilst on anti-TNF therapy due to neurological and respiratory complications including inability to clear secretions. The clinicians felt this was due to NPC progression rather than anti-TNF treatment. However, this patient experienced an initial honeymoon period of improving motor skills, better respiratory function, nutrition, pain control (pain due to perianal disease and abscesses) during the first year of treatment with a significant improvement in quality of life. Patient 1 had an improvement in both a large perianal wound pocket and a vaginal fistula and became able to swallow small amounts again. Unfortunately, the patient did not have an endoscopy prior to starting treatment so it is unclear if this was due to improvement in neurological function or upper gastrointestinal manifestations of Crohn’s disease.

### Efficacy of anti-TNF treatment in NPC1 patients with Crohn’s disease like intestinal inflammation

Four patients had a partial response to infliximab therapy. One patient had poor disease control on infliximab. Despite this, infliximab appeared to achieve better reported disease control on clinician global assessment than prior immunosuppression, steroids and exclusive enteral nutrition.

**Figure 3.**
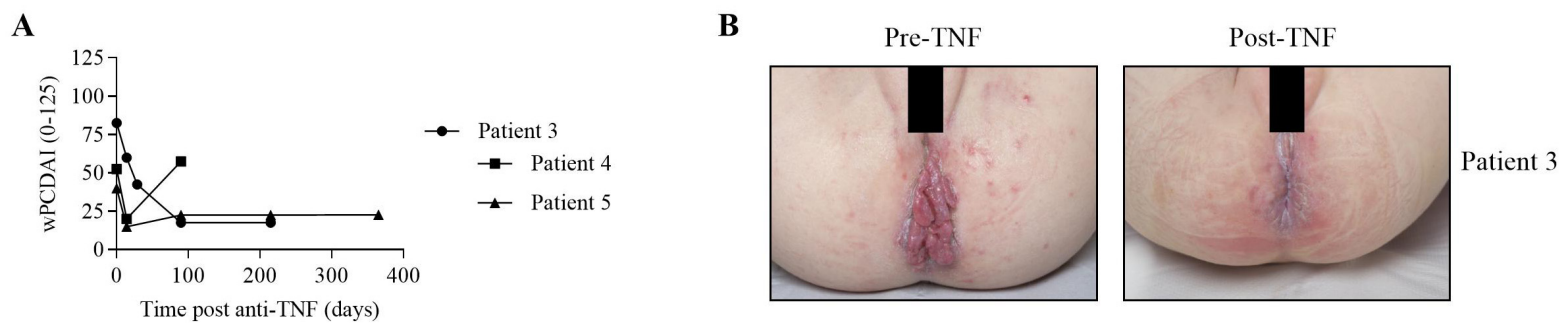
Clinical experience with anti-TNF (tumour necrosis factor) in Niemann-Pick Type C patients with inflammatory bowel disease (IBD). **A**: Disease activity over time in patients 2, 3 and 4. Time points “0” indicated baseline disease activity before start of infliximab. Disease activity was determined by mathematically weighted Pediatric Crohn’s Disease Activity Index (wPCDAI, range 0-125 points). **B**: Anti-TNF therapy results in marked improvement of severe perianal disease in patient 2.

We assessed patient response to treatment using the wPCDAI scoring system to indicate severity of intestinal symptoms at baseline, 4 weeks, 10 weeks, 6 months and a year. Patient 2 initially had severe Crohn’s disease with a wPCDAI of 82.5 and showed a significant improvement in score to 17.5 over the first 90 days of treatment with anti-TNF therapy ( Figure 3A). This improvement was maintained for a further 125 days whilst continuing anti-TNF therapy. There was also a marked improvement in perianal disease ( Figure 3B). Patient 3 initially improved wPCDAI scores from 52.5 to 20. However, this improvement was not maintained and the wPCDAI increased to 57.5 due to worsening abdominal pain, increased ESR and weight loss. Patient 4 had an initial score of 40 improving to 15 after 2 weeks before increasing to 22.5 from week 14 to 52 (due to increase in stool frequency).

## Discussion

NPC1 patients can present with severe Crohn’s-like disease in particular debilitating perianal disease. Anti-TNF therapy in NPC1-IBD patients results in symptom improvement without neurological deterioration at 1 year of follow up. We show in an *in-vitro* cell model increased levels of TNF upon LPS and bacterial stimulation suggesting that TNF is a differentially expressed cytokine in NPC1 and providing a molecular rationale of the treatment.

Patients with NPC1 suffer from a severe life-limiting disease, the potential risks of TNF therapy (demyelination and increased infection susceptibility) must be balanced against the improvement in quality of life due to effective IBD therapy and symptom control. Our data has shown improved management of Crohn’s-like disease in patients with NPC following anti-TNF therapy without worsening neurological function or infectious complications. Our data suggest that anti-TNF is safe to use in NPC from a neurological perspective. The improvement in IBD activity in NPC1 patients ( Figure 3) was associated with reduced pain, perianal disease and improved nutritional status. This suggests an improved quality of life for these patients. The response rate of anti-TNF in NPC1 patients is not complete and not sustainable (secondary loss of response) but very similar to patients with classical severe therapy-refractory Crohn’s disease ^14^.

TNF can promote neuro-inflammation and neuronal damage as well as having a protective role in some diseases (such as ADA2 deficiency ^15^) ^16^. Elevated levels of TNF within demyelinating plaques has been associated with the development of multiple sclerosis (MS) ^17^. Polymorphism in both TNFR1 and TNFR2 have been linked to an increased risk of MS ^18, 19^. Similarly, anti-TNF therapies have been linked to CNS demyelination and increased disease burden in patients with multiple sclerosis with a dose-dependent increase in relapse rate found in a trial of the TNF antagonist lenecept ^17^. Despite this, anti-TNF associated demyelination occurs independently to the classical risk factors for MS ^9^. Cases of anti-TNF associated demyelination have been described in patients with Crohn’s disease, psoriasis, rheumatoid arthritis and ankylosing spondylitis potentially hinting to a rare but relevant safety signal ^20– 22^. For example, in 75 patients starting anti-TNF therapy for rheumatoid arthritis or spondyloarthropathies, three patients who developed neurological symptoms demonstrated new cortical lesions consistent with demyelination on MRI ^6^. Another case series of four patients with neurological symptoms on anti-TNF therapy demonstrated MRI changes and CSF oligoclonal bands with three patients meeting the diagnostic criteria for multiple sclerosis ^23^. However, pathophysiological mechanisms behind these effects are not completely understood ^24^. Several mechanisms have been proposed, including poor cerebral penetration of anti-TNF agents due to large molecular size ^25^ which could prevent immunosuppressive actions in the brain. Another theory is based on the multiple forms and receptors for TNF. Soluble TNF signals through TNFR1 which is a ubiquitous receptor whose signalling can result in both pro and anti-apoptotic effects ^26^ but more commonly results in inflammation and increased apoptosis through caspase 8 activation ^27^. In contrast, signalling from transmembrane TNF is largely through TNFR2. TNFR2 is found on immune cells, endothelial cells and some cells of the CNS and is thought to be neuroprotective and stimulate cell survival through phosphatidylinositol 3-kinase (PI3K) activity ^26^. These opposing responses combined with variable receptor expression results in complex consequences of TNF manipulation and could explain why TNF may have both beneficial and deleterious role in multiple sclerosis ^24^.

NPC1 is a disorder of hypomyelination ^28^ and is characterised by progressive neurological decline. It is therefore important to ensure no worsening of neurological function in patients treated with anti-TNF. Unfortunately, it is difficult to formally assess cognitive function in this patient group in retrospect and therefore clinician reported outcome was used. In this cohort none of the patients experienced symptomatic episodes suggestive of demyelination or unexpected worsening of neurological function within a year of follow up. Anti-TNF therapy can also be associated with non-demyelinating neuroinflammation such as meningoencephalitis and vasculitis ^10^. Whilst these are rare events, we did not see any evidence of this in our cohort. The increased infection risk associated with anti-TNF therapy is also relevant in NPC1 patients, especially regarding respiratory infections, but there was no reported increase in infection rates ( Table 2). The improvement in intestinal disease activity in NPC1 patients ( Figure 3) was associated with reduced pain, perianal disease and improved nutritional status. This suggests an improved quality of life for these patients.

Limitations of this study include the small patient cohort and retrospective data collection from medical notes. Whilst a randomised, prospective study would be informative this would be very difficult to achieve given that NPC1 is a rare disease. As data was obtained from medical notes written before study recruitment, bias in data collection was reduced. However, as a result of this we had fewer variables that we could study, and this led to incomplete data collection for one patient. As a result of this missing data this patient was not included in data analysis.

Selection criteria included diagnosis of NPC1 with intestinal inflammation and treatment with anti-TNF agents. As a result, we did not expect or see changing eligibility over time.

In summary, our data advocates the use of anti-TNF therapy in the clinic to improve quality of life in patients with severe CD like intestinal inflammation in the context of NPC1. Our results in this high-risk group of patients complement previous findings that the incidence of adverse neurological effects associated with anti-TNF use is low in patients with paediatric IBD ^8^.

## Data availability

Zenodo: Anti-TNF therapy for inflammatory bowel disease in patients with neurodegenerative Niemann-Pick disease Type C. https://doi.org/10.5281/zenodo.5668321 ^12^.

Data are available under the terms of the Creative Commons Attribution 4.0 International license (CC-BY 4.0).
