# Supplementary material for: Anti-TNF therapy for inflammatory bowel disease in patients with neurodegenerative Niemann-Pick disease Type C
Source: Wellcome Open Res. 2022 Jan 11;7:11. [Version 1] doi: 10.12688/wellcomeopenres.16986.1 (PMC9171292; doi:10.12688/wellcomeopenres.16986.1)
Supplement: Peer review history

# Peer review: Reviewer response for version 1

**DOI:** 10.21956/wellcomeopenres.18754.r50669

**Published:** 2022-06-06

**Authors:** Salvador Soriano

**Affiliations:** 1 Department of Pathology and Human Anatomy, School of Medicine, Loma Linda University, Loma Linda, CA, USA

In this report, Williams *et al.* aim to investigate the safety and effectiveness of anti-TNF therapy in NPC patients with Crohn's disease symptoms. To do so, they evaluate retrospective data from four patients with confirmed NPC1 mutations and intestinal inflammation and provide *in vitro* evidence suggesting that TNF expression may be inherently higher in NPC.

 This is a well-written article, and I agree with the authors' conclusion that anti-TNF therapy appears to be safe in patients with NPC1 mutations and may be an effective strategy for the management of intestinal inflammation in these patients.

 I have a couple of suggestions:

 The authors show that the treatment of PBMCs with U18666A, which is a widely used approach to generate a chemically-induced *in vitro* model of NPC, does not increase TNF-alpha by itself (Figure 1A). They then quantify changes in TNF-a expression in response to bacterial exposure (Figures 1C, D), and show that the response of NPC-like cells is stronger than that of control cells. The authors suggest that this increase is consistent with an increased TNF production in response to LPS and gut bacteria contributing to mucosal inflammation in NPC patients. This, of course, would provide a mechanism for the reported improvement in quality of life in response to anti-TNF in the study patients. While I agree with the authors' overall interpretation of their data, I would suggest expanding the discussion to include the article by Iftakhar-E-Khuda *et al.* ^1^ who reported that another U18666A model does display TNF-a increase in the absence of bacterial challenges, albeit within a different time frame. How do those findings fit into the authors' conclusions?

 The Discussion incorporates published work on the use of anti-TNFa therapies in other diseases. I think it would also be appropriate to include citations reporting the effects of anti-TNFa therapy on NPC liver disease (i.e., Rimkunas *et a*l. ^2^, Vincent *et al.* ^3^), especially in view of the link between liver dysfunction and Crohn's disease.

Is the work clearly and accurately presented and does it cite the current literature?

Yes

If applicable, is the statistical analysis and its interpretation appropriate?

Yes

Are all the source data underlying the results available to ensure full reproducibility?

Yes

Is the study design appropriate and is the work technically sound?

Yes

Are the conclusions drawn adequately supported by the results?

Yes

Are sufficient details of methods and analysis provided to allow replication by others?

Yes

Reviewer Expertise:

Molecular mechanisms of pathogenesis in Niemann-Pick disease type C; Alzheimer's disease; Parkinson's disease.

I confirm that I have read this submission and believe that I have an appropriate level of expertise to confirm that it is of an acceptable scientific standard.

## References

[1] : Novel mechanism of U18666A-induced tumour necrosis factor-alpha production in RAW 264.7 macrophage cells. *Clin Exp Immunol* .2009;155(3) : 10.1111/j.1365-2249.2008.03779.x 552-8 10.1111/j.1365-2249.2008.03779.x 19220841PMC2669532

[2] : TNF-{alpha} plays a role in hepatocyte apoptosis in Niemann-Pick type C liver disease. *J Lipid Res* .2009;50(2) : 10.1194/jlr.M800415-JLR200 327-33 10.1194/jlr.M800415-JLR200 18815434PMC2636917

[3] : Evaluation of an anti-tumor necrosis factor therapeutic in a mouse model of Niemann-Pick C liver disease. *PLoS One* .2010;5(9) : 10.1371/journal.pone.0012941 e12941 10.1371/journal.pone.0012941 20886067PMC2944848

# Peer review: Reviewer response for version 1

**DOI:** 10.21956/wellcomeopenres.18754.r47995

**Published:** 2022-01-17

**Authors:** Marco Gasparetto

**Affiliations:** 1 Department of Paediatric Gastroenterology, The Royal London Hospital, Bart's Health Trust, London, UK

**Major comments**

- The authors present a case series of four paediatric patients with Type C1 Niemann-Pick disease and Crohn’s-like intestinal inflammation diagnosed between 2014 and 2020 treated with anti-TNF. They address the important question of the safety of anti-TNF treatments in patients affected by this neuro-disabling condition, in view of previous reports of demyelinating and non-demyelinating side effects of anti-TNF. The data supports the use of anti-TNF in patients with NPC1.
- Within the intrinsic limitations of a retrospective series of four cases, the findings described in this paper are relevant to the rare patients affected by NPC1 and who also present with IBD phenotype.
- The paper is well written. The introduction provides a good summary of the epidemiology data available and the pathophysiology underlying NPC1. The literature review is complete, with only one reference that may benefit from updating (see Minor comments).
- The case series is backed up by *in vitro* experiments on PBMCs from healthy donors, including small molecule inhibition of NPC1 and infection models.
- Results are described clearly.
- Tables and figures are helpful to the reader and complementary to the information provided in the text.
- The discussion is well articulated and leads to a relevant conclusive message.
- It would be relevant and interesting to examine infliximab level and anti-infliximab antibody level in the four patients described. Therapeutic drug monitoring would add further insight into how patients with NPC1 respond to infliximab. Three out of the four patients described had a partial response to IFX, and it’d be relevant to know whether loss of response was due to the development of anti-IFX antibody as opposed to low IFX trough levels. Is this data available and can it be analysed? If not, could the authors discuss whether anti-TNF therapeutic drug monitoring could/should be evaluated in future studies of patients with NPC1 and IBD?

**Minor comments**

- Methods, Page 4 – Statistical analysis: “R is an open access alternative” – Can the authors clarify whether R was utilised or not for data analysis in this specific work?
- References: *[5] Ruemmele FM, Veres G, Kohlo KL, et al. Consensus guidelines of ECCO/ESPGHAN on the medical management of pediatric Crohn’s disease. J Crohns Colitis. 2014; 8(10): 1179-207*. These guidelines were updated in 2020 and you may want to add the following updated version:
  - [5] van Rheenen PF, Aloi M, Assa A, et al. The Medical Management of Paediatric Crohn's Disease: an ECCO-ESPGHAN Guideline Update. Journal of Crohns and Colitis 2020: 1-24 ^1^.

Is the work clearly and accurately presented and does it cite the current literature?

Yes

If applicable, is the statistical analysis and its interpretation appropriate?

Yes

Are all the source data underlying the results available to ensure full reproducibility?

Yes

Is the study design appropriate and is the work technically sound?

Yes

Are the conclusions drawn adequately supported by the results?

Yes

Are sufficient details of methods and analysis provided to allow replication by others?

Yes

Reviewer Expertise:

Paediatric gastroenterology with sub-specialist interest in paediatric IBD

I confirm that I have read this submission and believe that I have an appropriate level of expertise to confirm that it is of an acceptable scientific standard.

## References

[1] : The Medical Management of Paediatric Crohn's Disease: an ECCO-ESPGHAN Guideline Update. *J Crohns Colitis* .2020; 10.1093/ecco-jcc/jjaa161 10.1093/ecco-jcc/jjaa161 33026087
